# DNA-PKcs is required to maintain stability of Chk1 and Claspin for optimal replication stress response

**DOI:** 10.1093/nar/gku116

**Published:** 2014-02-05

**Authors:** Yu-Fen Lin, Hung-Ying Shih, Zengfu Shang, Shinji Matsunaga, Benjamin PC Chen

**Affiliations:** ^1^Division of Molecular Radiation Biology, Department of Radiation Oncology, University of Texas, Southwestern Medical Center at Dallas, Dallas, TX 75390, USA and ^2^Division of Molecular Pharmacology, Department of Pathophysiological and Therapeutic Science, Tottori University, Japan

## Abstract

The ataxia telangiectasia mutated and Rad3-related (ATR)-checkpoint kinase 1 (Chk1) axis is the major signaling pathway activated in response to replication stress and is essential for the intra-S checkpoint. ATR phosphorylates and activates a number of molecules to coordinate cell cycle progression. Chk1 is the major effector downstream from ATR and plays a critical role in intra-S checkpoint on replication stress. Activation of Chk1 kinase also requires its association with Claspin, an adaptor protein essential for Chk1 protein stability, recruitment and ATR-dependent Chk1 phosphorylation. We have previously reported that, on replication stress, the catalytic subunit of DNA-dependent protein kinase (DNA-PKcs) is rapidly phosphorylated by ATR at the stalled replication forks and is required for cellular resistance to replication stresses although the impact of DNA-PKcs onto the ATR signaling pathway remains elusive. Here we report that ATR-dependent Chk1 phosphorylation and Chk1 signaling are compromised in the absence of DNA-PKcs. Our investigation reveals that DNA-PKcs is required to maintain Chk1–Claspin complex stability and transcriptional regulation of Claspin expression. The impaired Chk1 activity results in a defective intra-S checkpoint response in DNA-PKcs–deficient cells. Taken together, these results suggest that DNA-PKcs, in addition to its direct role in DNA damage repair, facilitates ATR-Chk1 signaling pathway in response to replication stress.

## INTRODUCTION

ATR signaling through Chk1 is the major pathway activated in response to stalled replication forks or replication stress and the key regulator of the intra-S checkpoint (1,2). This checkpoint is required for the maintenance of genomic integrity to prevent genetic disorders, aging and carcinogenesis. It is generally agreed that, on replication stress, uncoupling of the Minichromosome maintenance (MCM) helicase complex and the replication machinery results in the formation of a long stretch of single-stranded DNA (ssDNA), which is quickly bound by RPA (3). Replication protein A (RPA)-coated ssDNA then recruits the ATR–ATR-interacting protein (ATRIP) complex and actives the ATR signaling pathway and its downstream phosphorylation targets including Chk1 kinase (4). ATR-mediated Chk1 phosphorylation at Ser317 and Ser345 stimulates Chk1 kinase and releases it from chromatin to carry out the intra-S checkpoint (5,6). The activity of Chk1 is also tightly associated with Claspin, an adaptor protein essential for recruitment of Chk1 and ATR-dependent Chk1 activation (7,8). Chk1 forms a stable complex with Claspin even in the absence of DNA damage and complex formation stabilizes both proteins (9–11). Furthermore, Claspin is known to interact with ATR in the absence of DNA damage and is associated with chromatin (8,12). On replication stress, Claspin is phosphorylated by ATR at Thr916 and phosphorylation at this site prevents Claspin from proteosome-mediated degradation, thus facilitating its interaction with Chk1 and leading to sustained Chk1 activation (9,13).

The catalytic subunit of DNA-dependent protein kinase (DNA-PKcs) is the key regulator of the nonhomologous end-joining (NHEJ) pathway of DNA double-strand break (DSB) repair (14). DNA-PKcs is activated on assembly with its DNA-binding partner Ku70/80 heterodimer at DSBs to form the DNA-PK holoenzyme essential for NHEJ-mediated DSB repair. In addition, we have reported that DNA-PKcs is involved in replication stress regulation and is phosphorylated by ATR at the Thr2609 cluster region on ultraviolet (UV) irradiation (15), which is known to stall replication fork and cause replication stress. Further, DNA-PKcs mutants lacking a functional Thr2609 cluster conferred UV sensitivity, suggesting that ATR-dependent DNA-PKcs phosphorylation is required for replication stress response. This is consistent with reports that DNA-PKcs participates in cellular resistance to UV irradiation (16,17), and that DNA-PKcs is required for RPA2 hyperphosphorylation on UV irradiation (18). The involvement of DNA-PKcs in replication stress response was further demonstrated by the DNA-PKcs ‘3A’ knockin mutant mice, which harbor three alanine substitutions at the mouse DNA-PKcs Thr2605 cluster (human Thr2609 cluster) and develop congenital bone marrow failure and premature lethality due to impaired proliferation and excessive DNA damage accumulation in the hematopoietic stem cells (19). Embryonic fibroblasts derived from DNA-PKcs 3A mice were hypersensitive to various replication stress agents and are defective in multiple DNA repair pathways.

It has also been reported that DNA-PKcs interacts directly with Chk1 kinase, and their association facilitates DNA-PKcs function in NHEJ-dependent DNA end-joining (20), and that DNA-PKcs preserves continuation of DNA replication and prevents DSBs accumulation in the presence of DNA polymerase inhibitor aphidicolin (21). Therefore, we hypothesized that DNA-PKcs participates in the repair of stalled replication forks or in the ATR-Chk1 signaling pathway. To further elucidate the function of DNA-PKcs, we examined Chk1 kinase activation and intra-S checkpoint in different DNA-PKcs proficient and deficient cell lines. Here we report that DNA-PKcs regulates Chk1–Claspin complex stability and ATR-dependent Chk1 activation. Our data thus suggest that DNA-PKcs is required for optimal activation of ATR-Chk1 signaling and intra-S checkpoint in cellular response to replication stress.

## MATERIALS AND METHODS

### Cell culture and small interfering RNA transfection

Human cervical cancer HeLa cells, human colorectal carcinoma HCT116 cells and HCT116 DNA-PKcs^−/−^ cells (22) were maintained in α-minimum essential medium (MEM) supplemented with 10% fetal bovine serum and penicillin/streptomycin. hTERT immortalized human fibroblast lines including wild type (48BR), ATR hypomorphic (F02-98) (23) and DNA-PKcs hypomorphic (NM720) (24) were grown in α-MEM with 20% fetal bovine serum. Retroviral vector-mediated expression of small hairpin RNA against ataxia telangiectasia mutated (ATM) was performed as previously described (25). In brief, HeLa cells were infected with previously 293 T-packaged retroviral particles and selected with 10 µg/ml puromycin to achieve a stable cell line. The small hairpin green fluorescent protein plasmid was used as a control. Small interfering RNA (siRNA) oligonucleotides designed against DNA-PKcs (26) were transfected with RNAiMax (Invitrogen) as described previously (15).

### Western blot and immunofluorescence staining

Whole-cell lysate was prepared with lysis buffer (50 mM Tris 7.5, 0.2 M NaCl, 1% Tween-20, 1% NP40, 1 mM sodium orthovanadate, 2 mM β-glycerophosphate, 1 mM dithiothreitol and protease inhibitors) and then subjected for western blotting. For immunofluorescence staining, cells were fixed with 4% paraformaldehyde (PFA) in phosphate buffered saline (PBS) for 15 min, treated with sucrose buffer (0.5% Triton X-100, 20 mM HEPES, pH 7.5, 50 mM NaCl, 3 mM MgCl_2_ and 300 mM sucrose) for 5 min, and blocked with 5% normal goat serum in PBS for 1 h at room temperature. The cells were incubated with primary antibodies for 2 h, washed three times with PBS and then incubated with Alexa Fluor 488-anti-rabbit and Texas Red-conjugated anti-mouse secondary antibodies for 1 h (Invitrogen). Cells were then washed three times with PBS and mounted in Vectashield mounting medium with 4,6-diamidino-2-phenylindole (Vector Laboratories). For EdU labeling and RPA2 staining, cells were labeled with 50 µM EdU for 1 h and followed by 5 mM hydroxyurea treatment for 1 h. Cells were preextracted with cytoskeleton buffer (0.1% Triton X-100, 10 mM HEPES, pH 7.4, 0.1 M NaCl, 3 mM MgCl_2_ and 300 mM sucrose) and then fixed with 4% PFA. EdU was stained with Click-iT EdU imaging kit (Invitrogen). Counting of RPA2 foci was performed using FociCounter (SourceForge) software version 1.0. For immunostaining in a 96-well plate, cells were fixed and stained as described above. The nuclei were visualized by staining with 0.3% DAPI and were left in PBS. The images were scanned by IN Cell Analyzer 2000 (General Electric (GE)). Fluorescence intensity per area (density level) was calculated using IN Cell Analyzer 2000 software (GE) version 4-10590. DNA-PKcs mouse monoclonal antibody (NeoMarkers), anti-ATR, anti-ATM, anti-Claspin, anti-SMC1, anti-phosphor-S966 SMC1, anti-Rad17, anti-phospho-S645 Rad17, anti-Chk1, anti-phospho-S317 Chk1 (Cell Signaling), anti-RPA2, anti-phospho-S139 H2AX (Millipore) were commercially available from the indicated vendors.

### Flow cytometry analysis

To chase S-phase progress on replication stress, exponential growing cells were pulse-labeled with 50 µM EdU for 1 h and then treated with 5 mM hydroxyurea for 1 h. Cells were harvested at 3 and 7 h after releasing from hydroxyurea treatment and fixed with 4% PFA in PBS. Fixed cells were incubated with anti-phospho-Ser 10 Histone H3 antibody (Upstate) for 3 h at room temperature and then incubated with Alexa Fluor 488-anti-rabbit antibody. EdU was detected using Alexa Fluor 647 Click-iT EdU flow cytometry assay kit. Stained cells were resuspended in propidium iodide solution (0.1 mg/ml RNase A and 20 mg/ml PI in PBS) and analyzed using a FACSCalibur flow cytometer and CellQuest software (BD Biosciences).

### Protein stability assay

Cells were treated with 0.1 mg/ml cycloheximide (Sigma) and harvested at several time points. Whole-cell lysates were prepared as described as above and subjected for Immunoblotting analysis. Scanned images were quantified using ImageJ (NIH, USA) software. The half-life of the protein was calculated using the formula T_1/2_ = 0.3t/log^(D1/D2)^, where D1 and D2 correspond to the protein levels at two different times and t is the corresponding interval of time.

### Quantitative real-time polymerase chain reaction

Total RNA was extracted using an RNeasy Mini kit (Qiagen) according to the manufacturer’s instructions. First-strand cDNA was synthesized by reverse transcribing 1 µg of total RNA using a Protoscript M-MuLV *Taq* reverse transcriptase-polymerase chain reaction (RT-PCR) kit (New England BioLabs) according to the manufacturer’s instructions. For real-time PCR analysis, TaqMan Gene Expression Assays (Applied Biosystems) including Hs00898637_m1 for *CLSPN*, Hs00967506_m1 for *CHEK1* and Hs03003631_g1 for *18S rRNA* were used to detect gene expression levels. PCR mixture contained 2 µl of diluted cDNA (corresponding to 4 ng of RNA), 5 µl of 2× TaqMan Master Mix (Applied Biosystems) and 0.5 µl of gene-specific 20× TaqMan Gene Expression Assay in a final volume of 15 µl. Real-time PCRs were performed using Chromo 4 Real-Time PCR Detector and Opticon Monitor 3 (Bio-Rad Laboratories). The thermal cycling conditions were a 10-min denaturation at 95°C and followed by 40 cycles of 15 s at 95°C and 1 min 11 s at 60°C. Three replicates for each sample were analyzed using the 2^−^^ΔΔCT^ method (27).

### Fractionation

Soluble nuclear fraction and chromatin-bound fraction were prepared according to the fractionation protocol as described previously (28). Briefly, cell pellets were resuspended (2 × 10^7^ cells/ml) in buffer A (10 mM HEPES, pH 7.9, 10 mM KCl, 1.5 mM MgCl_2_, 0.34 M sucrose, 10% glycerol, 1 mM DTT, protease inhibitors and phosphatase inhibitors) containing 0.1% Triton X-100 and incubated for 5 min on ice. Nuclei (P1) was collected by low-speed centrifugation (1300 × *g*, 4 min, 4°C) and then washed once with buffer A. Washed nuclei were lysed with buffer B (3 mM EDTA, 0.2 mM EGTA, 1 mM DTT, protease inhibitors and phosphatase inhibitors) and incubated for 30 min on ice. Soluble nuclear fraction (S3) was separated from insoluble chromatin by centrifugation (1700 × g, 4 min, 4°C). The chromatin pellet was washed once with buffer B and centrifugated again. The final pellet (P3) was resuspended with Laemmli buffer and sonicated for 30 s.

### DNA fiber analysis

Cells were labeled with 100 µM iododeoxyuridine (IdU) for 10 min and then labeled with100 µM chlorodeoxyuridine (CldU) for 20 min for mock experiment. For monitoring DNA synthesis during replication stress, cells were labeled with IdU (100 µM) for 10 min, followed by exposure to CldU (100 µM) coupled with hydroxyurea (5 mM for HCT115 and 0.5 mM for human fibroblasts) for 1 h. DNA fibers were spread as described previously (29) and stained with primary antibodies (mouse anti-BrdU/IdU from BD Bioscience; rat anti-BrdU/CldU from Accurate Chemical) and fluorescence-conjugated secondary antibodies (Alexa Fluor 488-anti-rat and Texas Red-conjugated anti-mouse from Invitrogen). Fibers were imaged using Zeiss Axio Imager M2 and measured using AxioVision software (x64 version 4.8.3.0).

### Statistical analysis

Statistical analyses were performed using a two-way analysis of variance (ANOVA) followed by a Bonferroni’s post hoc test. All statistical analyses were performed using Prism GraphPad (version 6.02) software. Statistical significance was defined as *P* < 0.05, but values of *P* < 0.01, *P* < 0.001 and *P* < 0.0001 are shown as well to indicate level of confidence. The CldU to IdU ratios of DNA fiber tracks were first diagnosed the equal variance assumption by F-test, then test the significance by *t*-test.

## RESULTS

### DNA-PKcs is required for optimal Chk1 expression

**Figure 1.**
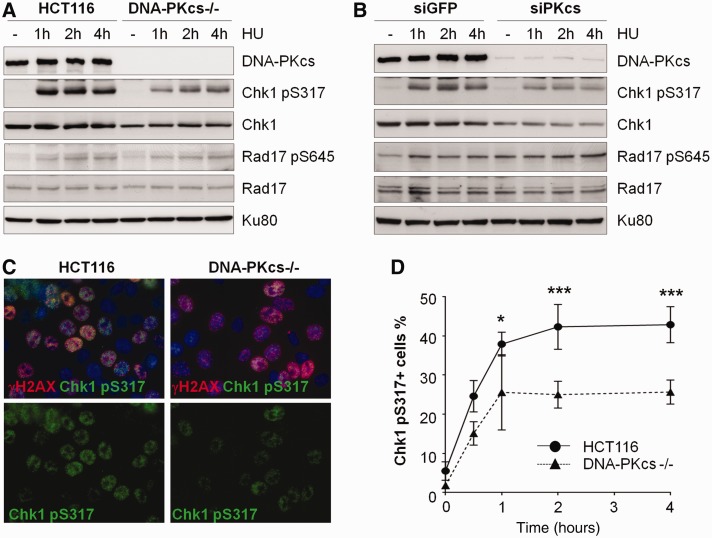
DNA-PKcs deficiency attenuates hydroxyurea-induced Chk1 phosphorylation. (**A**) The parental human HCT116 and DNA-PKcs^−/−^ cells were treated with 5 mM hydroxyurea (HU) and harvested at the indicated time points. Whole-cell lysates were analyzed by western blotting with the indicated antibodies. (**B**) HeLa cells transfected with siRNA against green fluorescent protein (siGFP) or DNA-PKcs (siPKcs) were western blot analyzed for HU-induced Chk1 phosphorylation. (**C**) HCT116 and DNA-PKcs^−/−^ cells were treated with 5 mM HU for 30 min and were immuno-stained for anti-γH2AX (red) and anti-phospho-S317 Chk1 (green) antibodies. (**D**) Cells were treated with HU for the indicated time durations. Percentages of Chk1 pS317 positive cells were scanned and quantified using In Cell 2000 Analyzer imaging system. The result was represented by mean values from three independent experiments. Statistical analyses were performed using two-way ANOVA. *P < 0.05; ***P < 0.001.

We have previously reported that, on UV-induced replication stress, DNA-PKcs is rapidly phosphorylated by ATR kinase at the T2609 cluster and such phosphorylation is required for cellular resistance to UV irradiation (15). However, the role DNA-PKcs or its phosphorylation status plays at stalled replication forks is not clear. To further investigate the significance of DNA-PKcs at replication associated damages, human colon cancer HCT116 cells and HCT116 DNA-PKcs knockout cells (abbreviated as DNA-PKcs^−/−^) were challenged with hydroxyurea-induced replication stress and were harvested at various time points afterward. Western blotting analysis revealed that ATR-mediated Chk1 phosphorylation at S317 (abbreviated as Chk1 pS317) was compromised in DNA-PKcs^−/−^ cells (Figure 1A). Chk1 pS317 reached the peak levels at 1–2 h and was sustained at 4 h in HCT116 cells, whereas its overall intensity was attenuated in DNA-PKcs^−/−^ cells. We noticed that the total protein levels of Chk1 were also decreased in DNA-PKcs^−/−^ cells. Similar defects in Chk1 pS317 and Chk1 protein expression were found in HeLa cells on siRNA-mediated depletion of DNA-PKcs (Figure 1B). Contrary to the defective Chk1 activation, ATR-dependent Rad17 phosphorylation and its expression were comparable in both DNA-PKcs proficient and deficient cells (with quantification analysis, data not shown), suggesting that DNA-PKcs is required for optimal Chk1 activation but not all ATR downstream signaling events (Figure 1A and B). These results were further substantiated by immunofluorescence staining that DNA-PKcs^−/−^ cells exhibited a decrease of Chk1 pS317 in response to hydroxyurea (Figure 1C) and UV irradiation (Supplementary Figure S1). Time course kinetics showed that ∼40% of HCT116 cells were positively stained for Chk1 pS317 at 1 h after hydroxyurea, whereas only ∼25% of DNA-PKcs^−/−^ cells were positive for Chk1 pS317 (Figure 1D). Thus, these results reveal that DNA-PKcs is required for optimal expression of Chk1.

### DNA-PKcs is required for the stability of Chk1–Claspin protein complex

**Figure 2.**
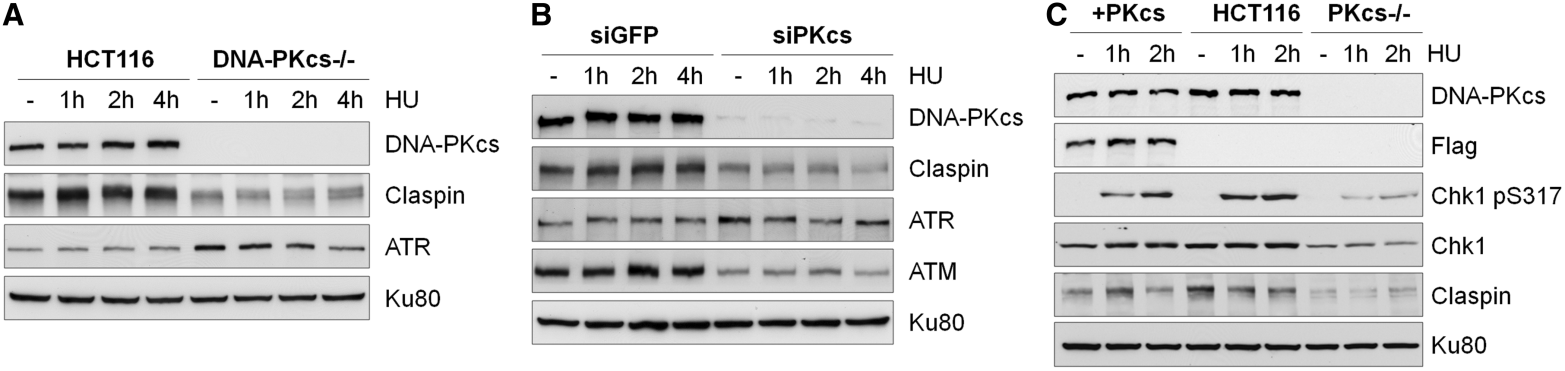
Decreased Claspin expression in DNA-PKcs–deficient cells. (**A**) HCT116 and DNA-PKcs^−/−^ cells were exposed to 5 mM HU for the indicated time points. Whole-cell lysates were analyzed with the indicated antibodies. (**B**) HeLa cells transfected with siRNA against GFP or DNA-PKcs were subjected to HU treatment and were analyzed as described above. (**C**) DNA-PKcs^−/−^ cells were complemented with a flag-tagged full-length DNA-PKcs (PKcs^+^). Expression of Chk1 and Claspin proteins as well as HU-induced Chk1 pS317 in PKcs^+^ cells were examined and compared with that in HCT116 and DNA-PKcs^−/−^ cells.

Chk1 forms a complex with the adaptor protein Claspin and their association is required for mutual stabilization of each other and Chk1 activation (8,9). Similarly, we observed that the decreased Chk1 activity correlated to the reduction of Claspin protein levels in DNA-PKcs–deficient cells. The steady state protein levels of Claspin were significantly reduced in DNA-PKcs^−/−^ cells (Figure 2A) and in HeLa cells on siRNA-mediated depletion of DNA-PKcs (Figure 2B). In the absence of DNA-PKcs, the expression of ATM was also attenuated (Figure 2B) as reported previously (26). To determine whether the reduction in Chk1–Claspin complex might be affected indirectly due to the decrease of ATM protein levels, HeLa cells expressing shRNA against GFP or ATM were subjected to hydroxyurea treatment. Western blotting showed that depletion of ATM did not affect Chk1–Claspin complex nor ATR-dependent Chk1 pS317 (Supplementary Figure S2A). Finally, we performed a rescue experiment to determine whether expression of DNA-PKcs could restore the Chk1–Claspin complex stability in DNA-PKcs^−/−^ cells. As shown in Figure 2C, expression of an exogenous flag-tagged DNA-PKcs was able to restore both Chk1 and Claspin protein levels as well as rescue HU-induced Chk1 S317 phosphorylation in DNA-PKcs^−/−^ cells as compared with HCT116 cells.

**Figure 3.**
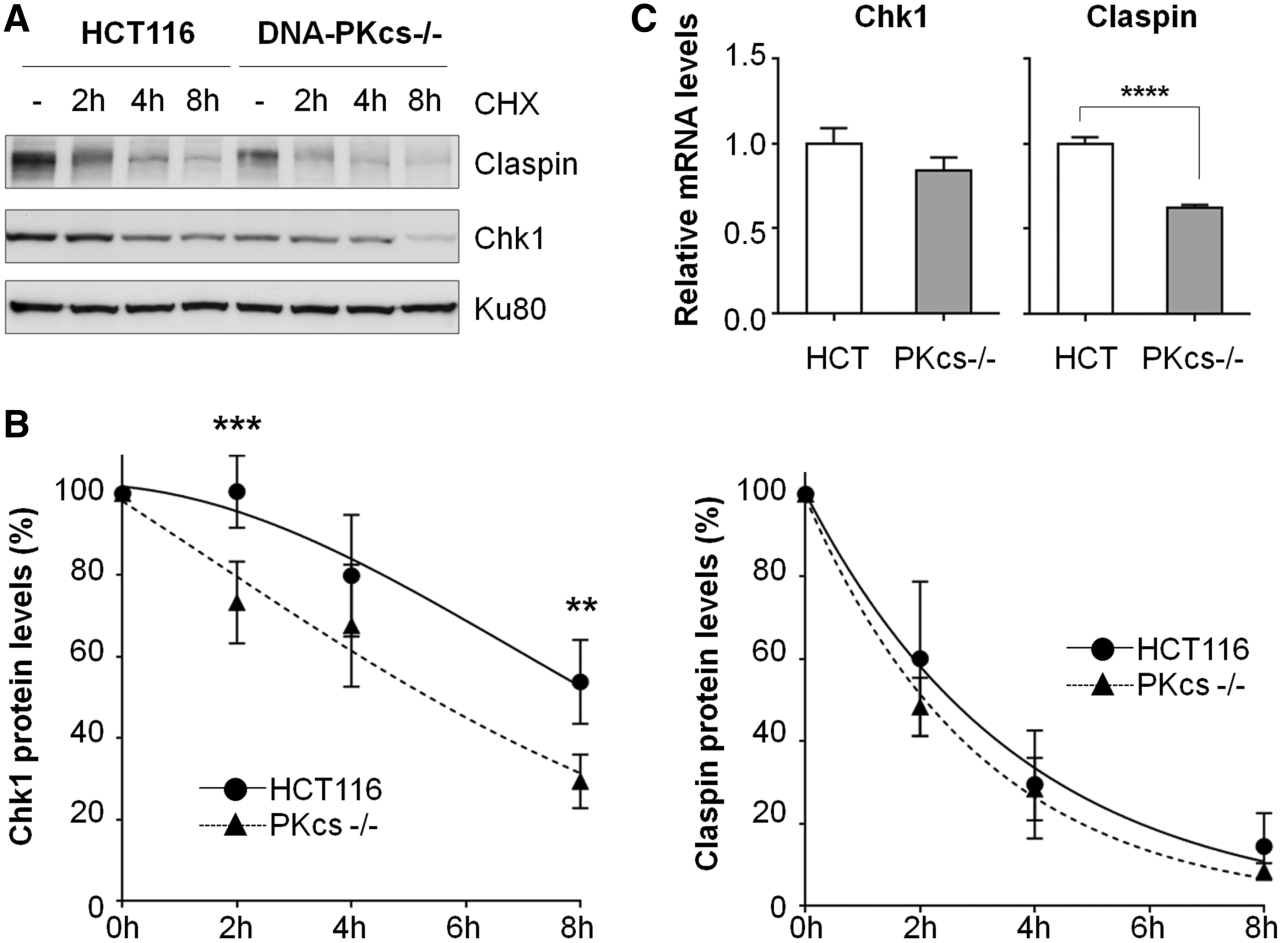
DNA-PKcs is required for *CLSPN* gene expression and Chk1 protein stability. (**A**) HCT116 and DNA-PKcs^−/−^ cells were incubated with protein synthesis inhibitor cycloheximide (CHX, 0.1 mg/ml) and were harvested at the indicated time points. Whole-cell lysates were subjected to western blotting analysis with anti-Claspin, anti-Chk1 and anti-Ku80 antibodies. (**B**) Total protein levels of Chk1 and Claspin before and after CHX treatment were quantified using ImageJ, and were normalized with Ku 80 protein levels. Relative Chk1 (left panel) and Claspin (right panel) protein stabilities were represented by mean values from at least three independent experiments. Statistical analyses were performed using two-way ANOVA. **P < 0.01; ***P < 0.001. (**C**) Steady-state mRNA levels of *CHEK1* and *CLSPN* genes in HCT116 and DNA-PKcs^−/−^ cells were measured by quantitative real-time PCR and normalized by *18S* rRNA level. Each bar represents the mean values from the three independent experiments. Statistical analyses were performed by Student’s t-test. ****P < 0.0001.

To further examine DNA-PKcs regulation on Chk1–Claspin stability, HCT116 and DNA-PKcs^−/−^ cells were treated with cycloheximide to block de novo protein synthesis and were harvested at various time points afterward to determine the steady state levels of Chk1 and Claspin proteins (Figure 3A). Quantification of western blot signals revealed that Chk1 protein was degraded rapidly in the absence of DNA-PKcs (Figure 3B). The half-life of Chk1 protein was 8.57 ± 1.72 and 4.99 ± 1.14 h in DNA-PKcs proficient and deficient cells, respectively. On the contrary, Claspin protein displayed similar half-life in DNA-PKcs proficient (2.25 ± 0.31 h) and in DNA-PKcs^−/−^ cells (2.03 ± 0.45 h). Though our result indicated that DNA-PKcs does not affect Claspin protein stability, steady state protein levels of Claspin were consistently lower in DNA-PKcs–deficient cells. Therefore, it is possible that DNA-PKcs might affect Claspin expression through transcriptional regulation similar to ATM expression in DNA-PKcs–deficient cells (26). To test this possible scenario, total RNA isolated from HCT116 and DNA-PKcs^−/−^ cells were analyzed by semiquantitative real-time PCR. As shown in Figure 3C, the expression of Claspin mRNA was significantly reduced in DNA-PKcs^−/−^ cells than in HCT116 cells, whereas Chk1 mRNA levels were similarly detected in both cells. Furthermore, overexpression of Claspin was able to improve the steady state protein levels of Chk1 in DNA-PKcs^−/−^ cells (Supplementary Figure S2B). Taken together, these results suggest that DNA-PKcs modulates the levels of Chk1 and Claspin through protein stability and transcriptional regulation, respectively.

### DNA-PKcs facilitates Chk1–Claspin complex formation and chromatin association

**Figure 4.**
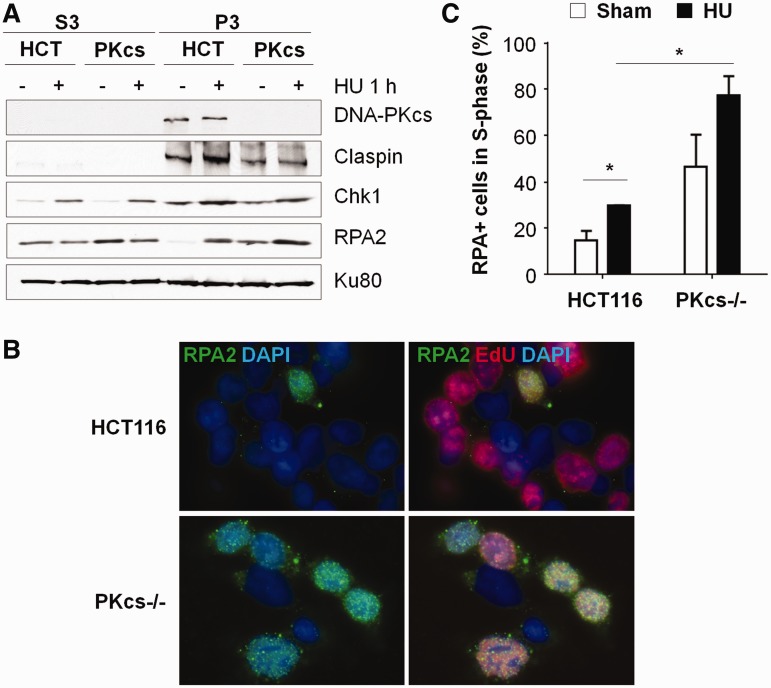
DNA-PKcs enhances Chk1 and Claspin association with chromatin on hydroxyurea treatment. (**A**) HCT116 and DNA-PKcs^−/−^ cells were subjected to 5 mM HU for 1 h. Soluble nuclear protein fraction (S3) and chromatin-nuclear matrix fraction (P3) were prepared for western blot analysis using antibodies against Claspin, Chk1, RPA2 and Ku80. (**B**) Increase of chromatin-bound RPA2 in DNA-PKcs^−/−^ cells. HCT116 and DNA-PKcs^−/−^ cells were pulse-labeled with 50 μM EdU for 1 h. Cells were preextracted with 0.1% TX-100 followed by immuno-staining with anti-RPA2 antibody (green) and EdU staining for S-phase cells (red). (**C**) Percentages of RPA2 positive cells among EdU-labeled S-phase cell population in the presence or absence of hydroxyurea. The result was generated from two independent experiments. Statistical analyses were performed using *t*-test. *P < 0.05.

Chromatin association of Claspin in response to replication stress is required for Chk1–Claspin complex formation and Chk1 phosphorylation (30). To examine whether DNA-PKcs affects chromatin association of the Chk1–Claspin complex, HCT116 and DNA-PKcs^−/−^ cells were treated with hydroxyurea and were subjected to chromatin fractionation (28). Claspin was bound to chromatin even before hydroxyurea treatment and addition of hydroxyurea further enhanced chromatin association of Claspin in HCT116 cells (Figure 4A). In the absence of DNA-PKcs, chromatin-bound Claspin was reduced owing to the decreased Claspin expression and hydroxyurea treatment did not further increase chromatin retention of Claspin (Figure 4A). Contrary to the decrease in chromatin-associated Claspin, there was an increase of RPA2 in chromatin fraction of DNA-PKcs^−/−^ cells before hydroxyurea treatment (Figure 4A). This notion was further supported by immunofluorescent staining that there was an increased frequency of chromatin-bound RPA2 among DNA-PKcs^−/−^ cells even in the absence of exogenous genotoxic stress (Figure 4B and C). These results suggest that there is a spontaneous increase of replication stress response in the absence of DNA-PKcs.

### DNA-PKcs is required for ATR-Chk1–dependent intra-S checkpoint response

**Figure 5.**
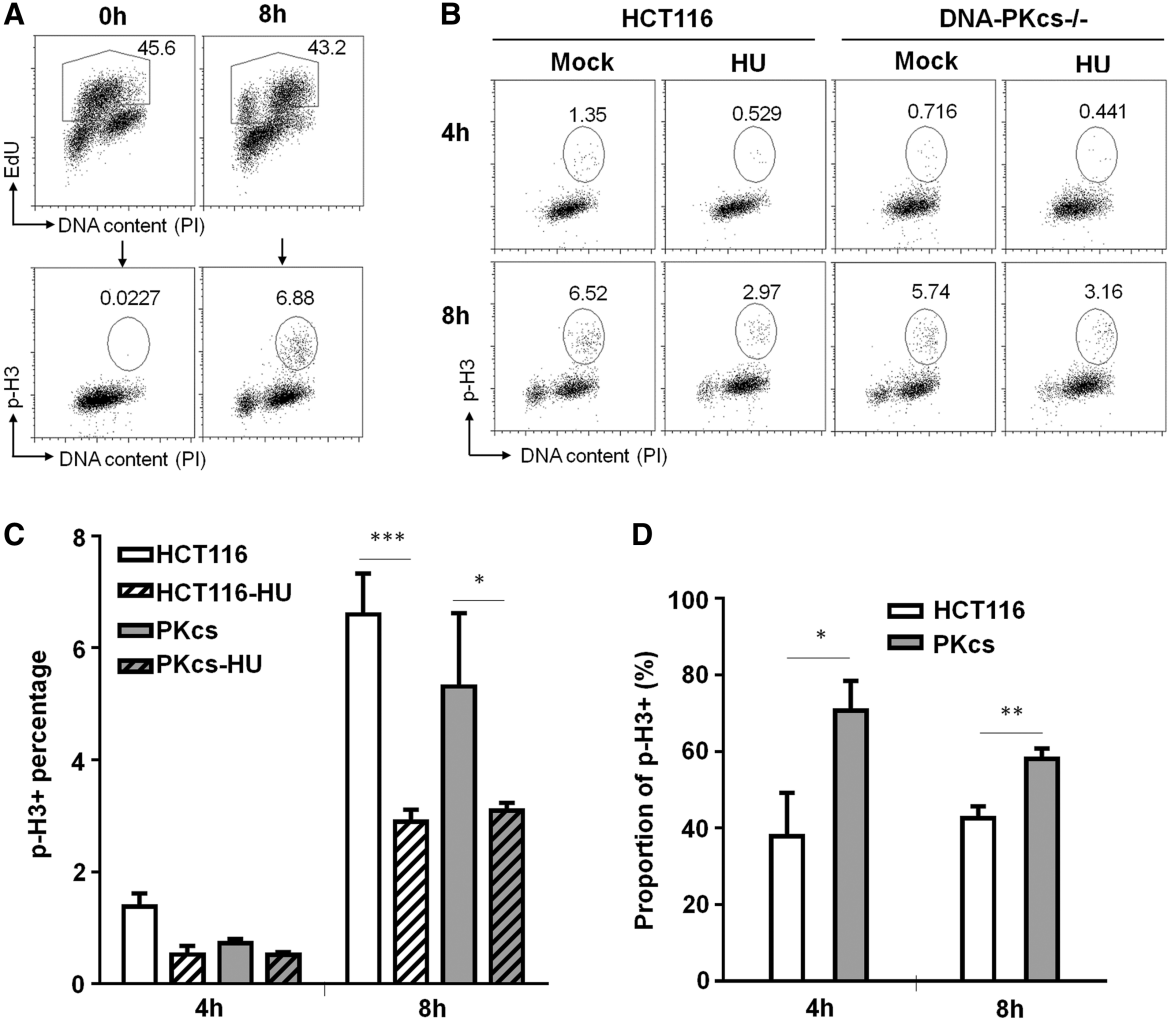
Attenuation of intra-S checkpoint in DNA-PKcs–deficient cells. (**A**) HCT116 and DNA-PKcs^−/−^ cells were pulse-labeled with EdU (50 µM) for 1 h, treated with 5 mM HU for 1 h and harvested at 4 and 8 h after HU treatment. Cell cycle progression of EdU-labeled cells were monitored for phospho-histone H3 (p-H3) by FACS analysis. The gating heptagons in the upper panel represent EdU(+) cells, which were further analyzed for p-H3(+) in the lower panel. (**B**) Representative images of p-H3 analysis. The gating ovals indicate the percentages of p-H3(+) among the EdU(+) cells at 4 and 8 h after HU treatment. (**C**) Percentages of EdU(+) cells migrated into mitosis (p-H3+) at 4 and 8 h. The result was generated from three independent experiments. (**D**) Proportions of pH3(+) in HU-treated samples compared with p-H3(+) in mock-treated samples. Statistical analyses were performed using *t*-test. *P < 0.05; **P < 0.01; ***P < 0.001.

The attenuation in Chk1 activation in DNA-PKcs–deficient cells suggested that DNA-PKcs could play a role in the intra-S checkpoint response. To further investigate this possibility, HCT116 and DNA-PKcs^−/−^ cells were pulse-labeled with EdU followed by hydroxyurea treatment for 1 hr. Progression of EdU-labeled S-phase cell populations were monitored for the expression of phospho-histone H3 (p-H3, a consensus maker for mitosis) by flow cytometry at different time points after hydroxyurea treatment (Figure 5). Our tracking of EdU(+) cells progression revealed that, in the absence of hydroxyurea, there was a slight reduction of p-H3(+) in DNA-PKcs^−/−^ cells as compared with HCT116 cells (Figure 5B and C). This indicates that DNA-PKcs^−/−^ cells progress through S phase slower than wild-type cells under normal and undisturbed condition. On hydroxyurea treatment, there was a significant reduction of p-H3(+) in both HCT116 and DNA-PKcs^−/−^ cells due to the intra-S checkpoint regulation. However, this intra-S checkpoint response was compromised in DNA-PKcs^−/−^ cells, as the relative proportions of p-H3(+) in hydroxyurea-treated samples verse sham controls (HU:Sham ratio) were significantly higher in DNA-PKcs^−/−^ cells (Figure 5D).

**Figure 6.**
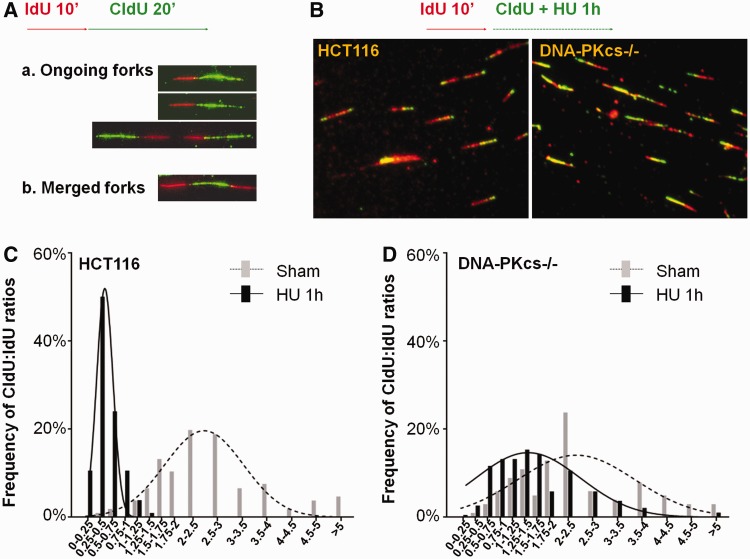
Defect in Chk1-mediated replication checkpoint in DNA-PKcs–deficient cells. (**A**) HCT116 and DNA-PKcs^−/−^ cells were pulse-labeled with iododeoxyuridine (IdU, 100 µM) for 10 min followed by chlorodeoxyuridine (CldU,100 µM) for 20 min. DNA tracks labeled with IdU (red) and CldU (green) were detected using monoclonal mouse and rat anti-BrdU antibodies, respectively. (**B**) HCT116 and DNA-PKcs^−/−^ cells were pulse-labeled with IdU for 10 min and followed by CldU labeling in the presence of 1 mM HU for 1 h. (**C** and **D**) The ratios of CldU to IdU in length were calculated from ongoing replication tracks (red-green tracks, N > 100). The profiles in the absence or presence of HU were analyzed in HCT116 cells (C) and DNA-PKcs^−/−^ cells (D).

Activation of ATR-Chk1 signaling pathway also attenuates the progression or velocity of active replication forks (31). The involvement of DNA-PKcs in Chk1-dependent intra-S checkpoint was further examined by the DNA fiber assay, in which the progress of ongoing DNA replication tracts can be monitored with sequential labeling with thymidine analogues iododeoxyuridine (IdU) and chlorodeoxyuridine (CldU) (Figure 6A). Our analysis revealed that normal DNA replication or progression of DNA replication forks was similar in HCT116 cells and DNA-PKcs^−/−^ cells (Figure 6C and D, and Supplementary Figure S3) in the absence of hydroxyurea (*P* = 0.169). The average ratios of CldU (20-min labeling) against IdU track (10-min labeling) were 2.60 ± 1.42 and 2.33 ± 1.39 in HCT116 and DNA-PKcs^−/−^ cells, respectively. On hydroxyurea treatment, the progression of replication was significantly attenuated due to ATR-Chk1–dependent checkpoint regulation. However, this replication checkpoint response was compromised in DNA-PKcs^−/−^ cells (Figure 6B). The average ratios of CldU track (1-h labeling in the presence of hydroxyurea) versus IdU track (10-min labeling) were 0.50 ± 0.26 in HCT116 cells and 1.12 ± 0.91 in DNA-PKcs^−/−^ cells (*P* < 0.0001). Taken together, the DNA fiber and fluorescence-activated cell sorting (FACS) analyses consistently indicate a defective intra-S checkpoint regulation in DNA-PKcs^−/−^ cells.

### Attenuation of Chk1–Claspin complex and intra-S checkpoint response in hypomorphic DNA-PKcs mutant cells

**Figure 7.**
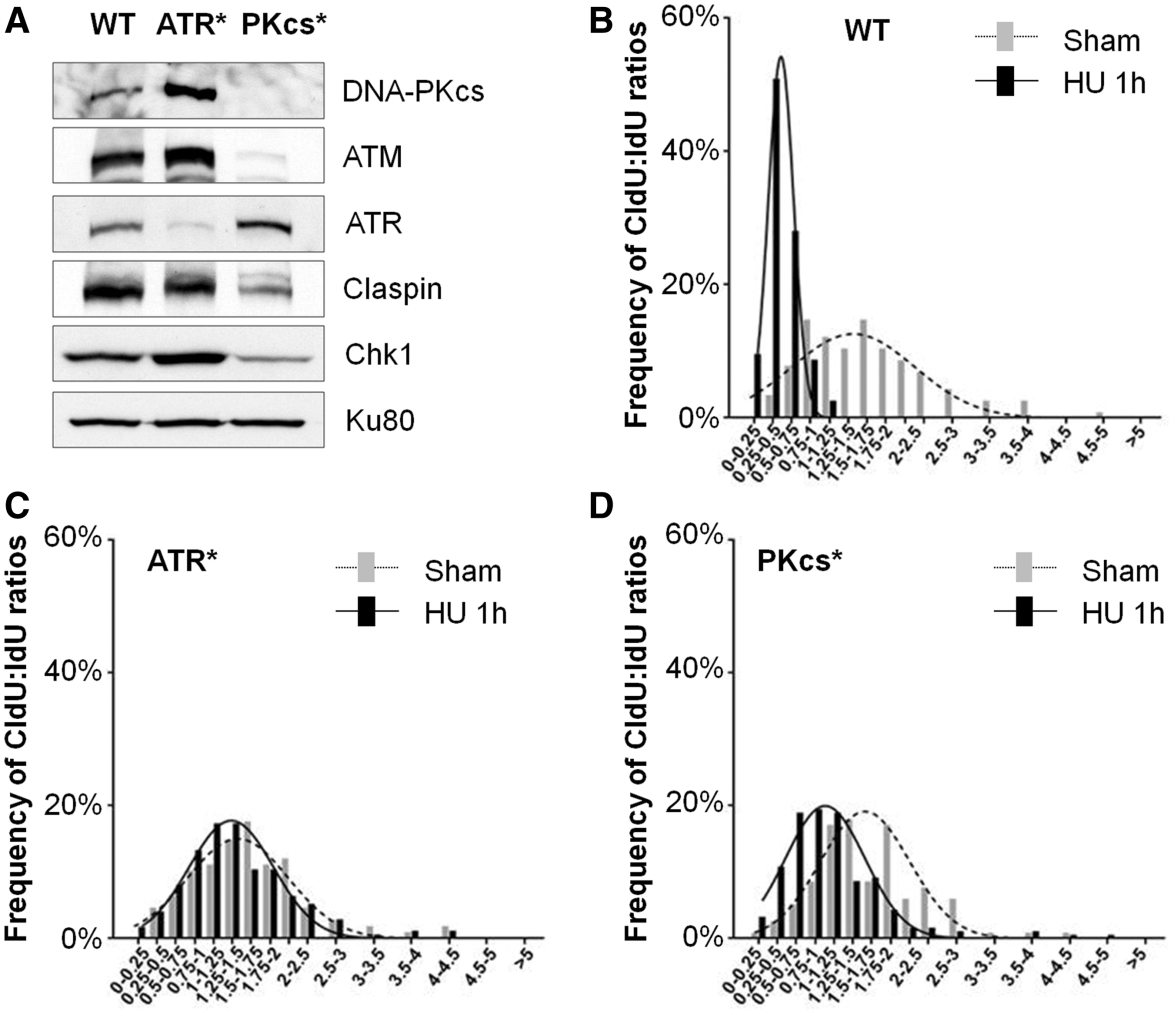
Defect of Chk1–Claspin stabilities and replication checkpoint in human DNA-PKcs hypomorphic fibroblasts. (**A**) Whole-cell lysates of control human fibroblasts (WT), ATR hypomorphic (ATR*) and DNA-PKcs hypomorphic (PKcs*) fibroblasts were western blot analyzed with the indicated antibodies. (**B**) Human wild type, ATR*, PKcs* fibroblasts were pulse-labeled with IdU (red) for 10 min and followed by CldU (green) labeling in the presence of HU (0.5 mM) for 1 h. (**C**) The ratios of CldU to IdU in length were calculated from ongoing replication tracks in WT, ATR* and PKcs* fibroblasts in the absence or presence of HU. The results were generated from >100 ongoing DNA replication tracks from each cell line.

Hypomorphic mutations in ATR and ATM genes have been identified among patients carrying Seckel syndrome and ataxia-telangiectasia (A-T), respectively. Hypomorphic mutations in DNA-PKcs encoding *RPKDC* gene have also been identified in many mammals but not in humans until recently (24,32). Woodbine *et al.* (24) reported a true hypomorphic mutation in human *RPKDC* gene and resulted in early lethality and neurological abnormalities. Consistent with our analysis in HCT116 DNA-PKcs^−/−^ and DNA-PKcs–depleted HeLa cells, we observed reductions of both Chk1 and Claspin proteins in DNA-PKcs hypomorphic mutant cells (Figure 7A), suggesting that the presence of DNA-PKcs is genuinely required for optimal expression and/or stabilization of the Chk1–Claspin protein complex. Furthermore, a similar defect in the replication-associated checkpoint was also found in DNA-PKcs hypomorphic cells when challenged with hydroxyurea treatment (Figure 7B–D). DNA fiber analysis revealed that the average ratios of CldU (20 min) to IdU (10 min) were 1.59 ± 0.78 in wild type control fibroblasts, 1.58 ± 0.73 in ATR-deficient Seckel cells (ATR*) and 1.58 ± 0.69 in DNA-PKcs hypomorphic cells (DNA-PKcs*) under normal condition. This data indicate the progression of replication forks is similar among three cell lines (WT versus ATR*: *P* = 0.867; WT versus DNA-PKcs*: *P* = 0.860; ATR* versus DNA-PKcs*: *P* = 0.999). On hydroxyurea treatment, the average ratios of CldU (1 h) to IdU (10 min) was significantly decreased in control fibroblasts (0.48 ± 0.21) but not in Seckel cells (1.44 ± 0.72) nor in DNA-PKcs hypomorphic cells (1.09 ± 0.68). Seckel cells exhibited similar distribution of CldU:IdU ratio under normal or hydroxyurea treatment. DNA-PKcs hypomorphic cells displayed compromised shift of distribution profile in the presence of hydroxyurea similar to that of ATR-deficient Seckel cells and is consistent with the DNA fiber analysis in HCT116 DNA-PKcs^−/−^ cells. These results further indicate that intra-S checkpoint regulation is also compromised in human DNA-PKcs hypomorphic cells.

## DISCUSSION

DNA-PKcs has been well characterized for its role in DSB repair and as a key regulator of the NHEJ pathway (33). In addition to DSB repair, several lines of evidence also suggested that DNA-PKcs plays an important role in replication-associated DSB repair or the replication stress response. For example, DNA-PKcs is required for cellular resistance to UV irradiation (16,17), which induces DNA replication blocking photolesions and activates the ATR signaling pathway to preserve and repair the stalled replication forks (1). In addition, DNA-PKcs has also been found to interact with RPA protein complex directly (34) and plays a key role in RPA2 phosphorylation (34,35). The direct evidence also came from our previous report that DNA-PKcs interacts with ATR kinase and is phosphorylated at the T2609 cluster region by ATR kinase on replication stress (15). Furthermore, DNA-PKcs^3A^ mutant mice lacking phosphorylation residues at the T2605 cluster (human T2609) conferred early lethality and congenital bone marrow failure. Further investigation revealed that cells derived from homologous DNA-PKcs^3A^ mice were highly sensitive toward replication stress agents and impaired in homologous recombination and Fanconi Anemia pathways essential for the repair of replication associated DNA lesions (19).

We hypothesize that DNA-PKcs being associated and phosphorylated by ATR kinase might in turn result in DNA-PKcs modulating ATR and downstream signaling events to facilitate the recovery of stalled replication forks or the repair of replication-associated DSBs. In the current study, we report that DNA-PKcs is required for preservation and stabilization of the Chk1–Claspin complex, and optimal ATR-Chk1 signaling in intra-S checkpoint regulations. The role of DNA-PKcs in stabilization of the Chk1–Claspin complex partly is due to transcriptional regulation of *CLSPN* gene as shown by real-time PCR analysis (Figure 3C). Involvement of DNA-PKcs in transcriptional regulation has been documented on several genes including *ATM* (26), fatty acid synthase (36) and Autoimmune regulator–dependent tissue-restricted antigens (37). Although it is not clear how DNA-PKcs promotes *CLSPN* gene expression, DNA-PKcs–dependent phosphorylation participates in chromatin remodeling and recruitment of upstream transcriptional factors on the target genes (37,38). Decrease in Claspin overall protein level likely alters Chk1 protein stability and hastens its degradation in the absence of DNA-PKcs. As a consequence, ATR-Chk1–dependent intra-S or replication checkpoint regulation was compromised in DNA-PKcs–deficient cells as measured by the progression of S-phase cell population (Figure 5) and ongoing DNA replication tracks in the DNA fiber assay (Figures 6 and 7). It is interesting that the levels of ATR protein is actually increased in DNA-PKcs–deficient cells (Figures 2A and 7A), which is contrary to the decrease of ATM protein levels in these cells. The increase in ATR expression is likely required to compensate the loss of DNA-PKcs and as well as partially attenuated ATM levels. Conversely, we observed an increased DNA-PKcs expression in the ATR hypomorphic cells. It is likely that, under ATR hypomorphic condition, elevated expressions of DNA-PKcs and ATM (depending on DNA-PKcs) are necessary to compensate the loss of ATR and to sustain cellular survival.

The ATR-Chk1–dependent intra-S checkpoint regulation in part by phosphorylation and inhibition of Cdc25 phosphatase, which activate Cdk kinases for replication origin firing and cell cycle progression (39). In addition, activation of the ATR-Chk1 signaling is required to attenuate the rate of DNA synthesis although the molecular mechanism of this ‘replication elongation checkpoint’ is not fully understood (31). Using the DNA fiber assay to monitor the progression of ongoing replication tracks, our results indicated that active replication forks fail to halt on hydroxyurea treatment in DNA-PKcs–deficient cells and in ATR hypomorphic cells (Figures 6 and 7). This further confirms a defective response of the ATR-Chk1signaling pathway without DNA-PKcs. In addition to halting the progression of active replication forks on replication stress, DNA-PKcs might also play a role in preserving and recovering the stalled replication forks as we observed an increase of spontaneous RPA2 loading onto chromatin in non-treated DNA-PKcs^−/−^ cells but not in HCT116 cells (Figure 4). This notion is supported by the report that DNA-PKcs is required for cellular resistance against DNA polymerase inhibitor aphidicolin and repair of replication-associated DSBs by aphidicolin (21). In agreement, our analysis indicated that DNA-PKcs kinase activity facilitates continuation of DNA replication in the presence of aphidicolin, and that combination of aphidicolin and DNA-PKcs kinase inhibitor Nu7441 completely halt active DNA replication (data not shown).

Consistent results of a defective Chk1 signaling were generated not only from HCT116 derivative DNA-PKcs^−/−^ cells and in cells in which DNA-PKcs protein levels were knocked down via siRNAs, but also skin fibroblasts carrying a hypomorphic mutation in DNA-PKcs. Taken together, our results provide compelling evidence that DNA-PKcs is required for optimal ATR-Chk1 signaling in the cellular response to replication stress. Similar to our findings, a recent study also suggested that DNA-PKcs could synergize ATR activation in a DNA structure-specific manner *in vitro*. Using a cell lysate–based assay and DNA template carrying a free DSB end and a short ssDNA gap, Vidal-Eychenie *et al.* (40) reported a robust activation of ATR-Chk1signaling *in vitro*, and that depletion or kinase inhibition of DNA-PKcs attenuated Chk1 activation, suggesting that DNA-PKcs could prime and trigger ATR-Chk1signaling in the presence of DSBs. This could reflect situations when active replication forks encounter DSBs directly (e.g. IR) or conversion of a single-strand break (e.g. Camptothecin) into a DSB due to ‘run-off’ of replication (34). It has also been suggested that this ‘run-off’ of replication or conversion of single-strand DNA lesions is the major source of endogenously occurred DSBs (41). Under this scenario, replication-associated DSBs trigger DNA-PKcs kinase and facilitate ATR-Chk1signaling pathway, although such DSBs will need to be further processed through ATM-dependent end resection procedure to produce ssDNA necessary to trigger ATR activation (42). One possible explanation is that DNA-PKcs–mediated RPA2 phosphorylation might facilitate Chk1 activation as RPA2 mutant lacking DNA-PKcs phosphorylation residues fails to support ATR-Chk1 signaling in response to various genotoxic stresses (43). However, replication stress does not always induce a rapid formation of DSBs. For example, UV or hydroxyurea treatment elicits a fast kinetic of ATR-Chk1 signaling, but a delay in replication-associated DSBs and DNA-PK kinase activation (44). Under such conditions, it is unlikely that DNA-PKcs kinase is necessary to promote the ATR-Chk1 signaling. Treatment with DNA-PKcs kinase inhibitor also did not affect Chk1–Claspin stability *in vivo* nor HU-induced Chk1 pS317 (data not shown).

ATR, ATM and DNA-PKcs are the three major kinases in response to various types of DNA damage. While hypomorphic mutations in *ATR* and *ATM* genes have been identified among patients carrying Seckel syndrome and A-T, respectively, little was known about mutations in DNA-PKcs encoding *RPKDC* gene in human until recently (24,32). A missense mutation (L3062R) was first identified from a patient with severe combined immunodeficiency (SCID) disorder. Although L3062R mutation conferred impaired DSB repair and radiosensitivity, it did not affect DNA-PKcs protein expression or its kinase activation (32). Woodbine *et al.*(24) reported the identification of the first true hypomorphic *RPKDC* in human with one Δexon16 mutation inactivated DNA-PKcs and one missense mutation (A3574V) led to a diminished but detectable residual activity of DNA-PKcs. The patient has barely detectable DNA-PK kinase activity, impaired DSB repair and SCID character as predicted. It was unexpected that the patient also displayed severe growth failure, microcephaly and global impairment in neurological function not found in other NHEJ-deficient patients. These features resemble those found in hypomorphic suppression of ATR in Seckel syndrome (23). Our analyses confirmed that hypomorphic DNA-PKcs diminished Chk1–Claspin complex and impaired ATR-Chk1 signaling in intra-S checkpoint responses (Figure 7). Thus the increased severity in DNA-PKcs hypomorphic patient than other NHEJ-deficient patients can be explained in part by the impaired ATR-Chk1 signaling pathway. Our investigation further demonstrates that the coordination and synergism between DNA-PKcs and ATR signaling is crucial for normal neurological development to counteract genotoxic stresses and to prevent pathophysiological progression.

In summary, we report here that DNA-PKcs is required for the maintenance of Chk1–Claspin complex stability and the optimal ATR-Chk1 signaling pathway. In the absence of DNA-PKcs, decrease of *CLSPN* gene transcript results and reduced Claspin protein levels lead to accelerated Chk1 protein degradation. As a result, ATR-Chk1–dependent intra-S cell cycle checkpoint regulation is compromised. Consistent results were generated from HCT116 DNA-PKcs^−/−^ cells and hypomorphic DNA-PKcs mutant cells from a SCID patient with severe neurological abnormalities. Further investigation of the cross talks between DNA-PKcs and ATR-Chk1 pathway is critical for elucidation the function of DNA-PKcs in replication stress responses as well as neurological development and brain tissue homeostasis.

## SUPPLEMENTARY DATA

Supplementary Data are available at NAR Online.

## FUNDING

National Institutes of Health (NIH) [CA166677] and the Cancer Prevention Research Institute of Texas [RP110465-P1]. Funding for open access charge: NIH [CA166677].

*Conflict of interest statement*. None declared.

## Supplementary Material

Supplementary Data
