# Supplementary material for: DNA-PKcs is required to maintain stability of Chk1 and Claspin for optimal replication stress response
Source: Nucleic Acids Res. 2014 Feb 5;42(7):4463–73. doi: 10.1093/nar/gku116 (PMC3985680; doi:10.1093/nar/gku116)
Supplement: Supplementary Data [file supp_42_7_4463__index.html]

DNA-PKcs is required to maintain stability of Chk1 and Claspin for optimal replication stress response — DNA-PKcs is required to maintain stability of Chk1 and Claspin for optimal replication stress response — Supplementary Data 

# DNA-PKcs is required to maintain stability of Chk1 and Claspin for optimal replication stress response

## Supplementary Data

files

**Files in this Data Supplement:**

- Supplementary Data - pdf file
