# Supplementary material for: DNA-PKcs is required to maintain stability of Chk1 and Claspin for optimal replication stress response
Source: Nucleic Acids Res. 2014 Feb 5;42(7):4463–73. doi: 10.1093/nar/gku116 (PMC3985680; doi:10.1093/nar/gku116)
Supplement: Supplementary Data [file supp_gku116_nar-02947-x-2013-File009.pdf]

Supplementary Figure 1

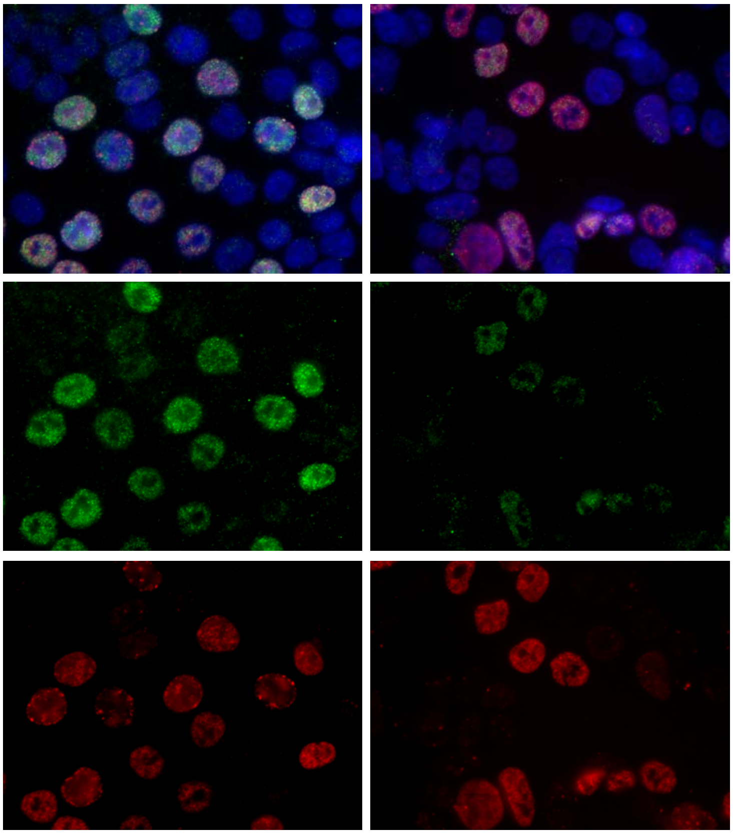

Supplemental Figure 1. HCT116 and DNA-PKcs$^{-/-}$ cells were subjected to UV irradiation (10 J/m^2), fixed at 30 min, and immunofluorescent stained with anti-γH2AX (red) and anti-pS317 Chk1 (green) antibodies.

Supplementary Figure 2

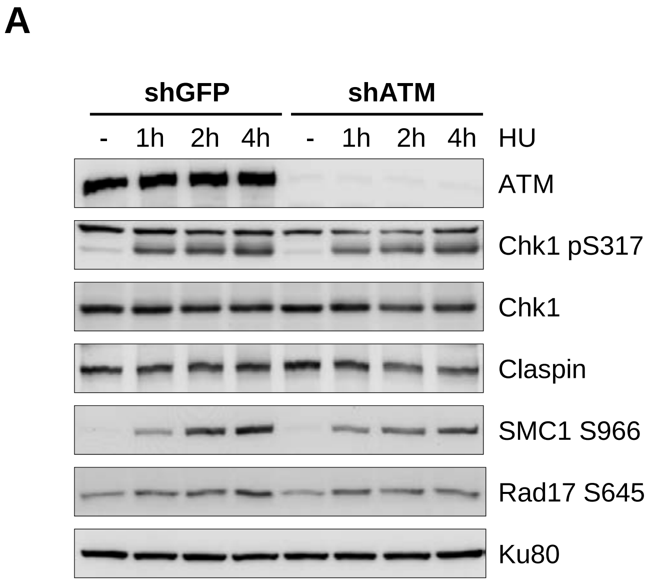

Supplemental Figure 2. (A) HeLa cells expressing small interfering hairpin RNA (shRNA) against GFP or ATM were subjected to 5mM HU treatment for the indicated time points. Whole cell lysates were analyzed with the indicated antibodies. The result showed that depletion of ATM did not affect Chk1-Claspin complex nor ATR-dependent Chk1 pS317. SMC1 S966 phosphorylation was partially affected in shATM cells due to the overlapping function between ATR and ATM kinases in SMC1 phosphorylation (Ref 44). (B) DNA-PKcs deficient HCT116 cells (DNA-PKcs-/-) were transfected with either empty vector (-) or a full length Claspin expression construct (Clp). Expression of Claspin in DNA-PKcs-/- cells improved the steady state protein levels of Chk1 in two independent experiments.

Supplementary Figure 3

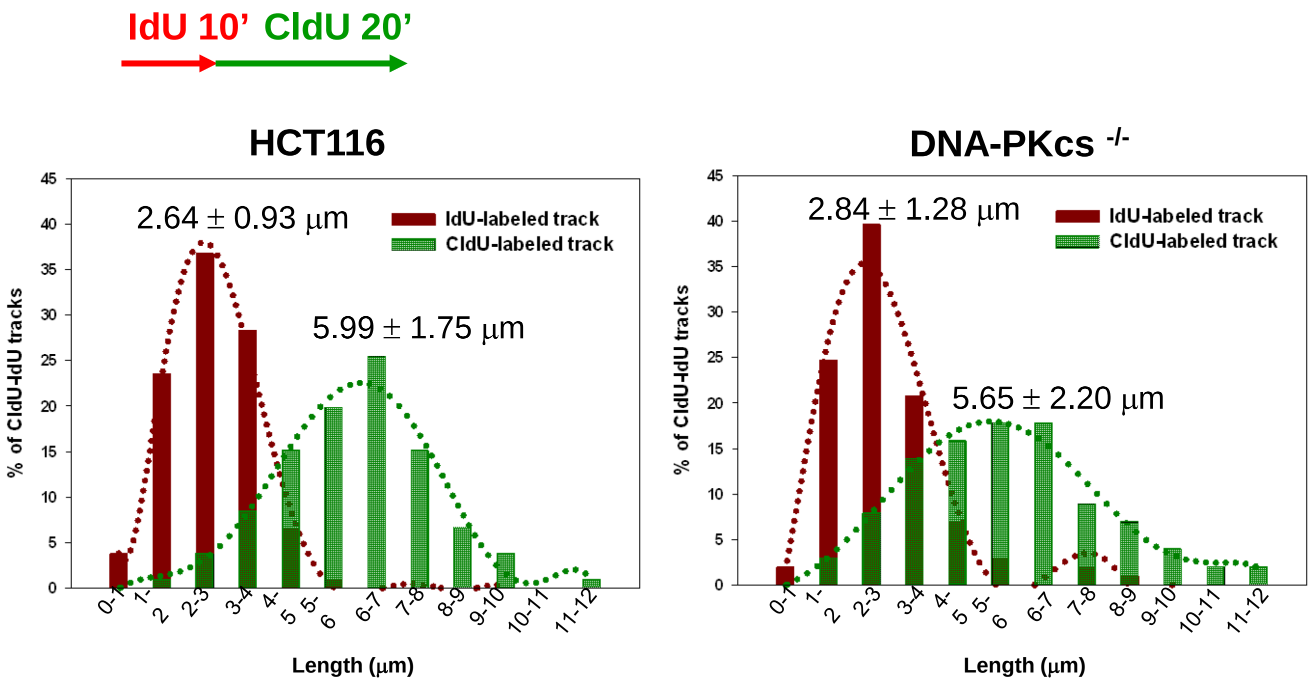

Supplemental figure 3. HCT116 and DNA-PKcs $^{-/-}$ cells were pulse-labeled with IdU (100 μM) for 10 min and CldU (100 μM) for 20 min sequentially. DN A replication tracks labeled with IdU and CldU were analyzed using indirect immunofluorescent with monoclonal mouse and rat anti-BrdU antibodies, respectively. The length of IdU- and CldU-labeled tracks were imaged in a Zeiss AxioImager microscope and were quantified using the AxioVision software. The numbers indicate mean values and standard deviations of each labeling. The results were generated from greater than 100 ongoing DNA replication tracks from each cell line.
